# Depressive symptoms as a mediator in the association between regular physical activity and irritable bowel syndrome-related symptom severity: a cross-sectional study among college students

**DOI:** 10.3389/fpsyg.2026.1856261

**Published:** 2026-09-15

**Authors:** Yuqian Sui, Ping Li, Xianfeng Yue, Yue Chen, Shiqing Feng, Jiajie Li, Ruihan Qi, Yixin Hu, Xinyan Wang, Jinxin Mei, Jianhong Qiao, Long Ji, Dong Li, Le Zhang, Xin Ye

**Affiliations:** 1 School of Public Health, Shandong First Medical University & Shandong Academy of Medical Sciences, Jinan, China; 2 Department of Nursing, The First Affiliated Hospital of Shandong First Medical University & Shandong Provincial Qianfoshan Hospital, Jinan, China; 3 Department of Health Management, Heze Medical College, Heze, China; 4 School of Clinical and Basic Medical Sciences, Shandong First Medical University & Shandong Academy of Medical Sciences, Jinan, China; 5 School of Nursing, Shandong Medical and Pharmaceutical University, Yantai, China; 6 School of Public Health, Jining Medical University, Jining, China

**Keywords:** anxiety, college students, depression, irritable bowel syndrome, mediation analysis, regular physical activity

## Abstract

**Introduction:**

Irritable bowel syndrome (IBS) is a common functional gastrointestinal disorder, substantially affecting quality of life among young adults. Although regular physical activity (RPA) benefits both mental and gastrointestinal health, its potential psychological mechanisms in IBS-related symptom severity remain unclear. This study was designed to examine the association between RPA and IBS-related symptom severity among Chinese college students and to clarify the mediating roles of depressive symptoms.

**Methods:**

A cross-sectional survey was conducted among 872 students from three universities in Shandong Province, China. Data on demographics, RPA, anxiety and depression assessed by the Self-Rating Anxiety Scale (SAS) and Self-Rating Depression Scale (SDS), and IBS-related symptom severity evaluated using the Irritable Bowel Syndrome Severity Scoring System (IBS-SSS) and the Gastrointestinal Symptom Rating Scale (GSRS) were collected. Group comparisons, multiple regression, and mediation analyses were performed.

**Results:**

Comparisons between students engaging in RPA and their sedentary peers revealed significantly lower IBS-SSS and GSRS scores in the RPA group. Consistent with these observations, multiple linear regression indicated that RPA was inversely associated with IBS-SSS [*β* = −28.665, 95% *CI*: (−40.129, −17.201), *p* < 0.01] and GSRS [*β* = −3.538, 95% *CI*: (−5.740, −1.335), *p* < 0.01] scores. In addition, higher SAS and SDS scores were positively associated with IBS-SSS and GSRS scores (all *p* < 0.001). Mediation analysis showed an estimated indirect association between RPA and IBS-SSS scores through depressive symptoms (*p* < 0.001), with a mediation proportion of 19.43%.

**Conclusion:**

These findings indicate that RPA is associated with reduced IBS-related symptom severity, and that depressive symptoms statistically account for part of this association, highlighting the importance of lifestyle strategies integrating physical and psychological interventions for managing IBS-related symptom in young adults.

## 1 Introduction

Irritable bowel syndrome (IBS), a common functional gastrointestinal disorder, is characterized primarily by abdominal pain closely associated with changes in bowel habits. Globally, IBS prevalence is estimated at approximately 10 to 15%, with incidence increasing annually (Arif et al., 2025; Black and Ford, 2020; Shrestha et al., 2022)_._ Long-term IBS increases the risk of developing chronic obstructive pulmonary disease, inflammatory bowel disease, and dementia (Porter et al., 2012; Chen et al., 2016; Lai et al., 2020).Although the pathogenesis of IBS remains incompletely understood, studies confirm its development is likely closely linked to small intestinal mucosal immune barrier function and micronutrient status (Ford et al., 2020; He et al., 2024; Worbs et al., 2006). Established risk factors include irregular dietary habits, high psychological stress, lack of exercise, family genetics, and environment (Das et al., 2024; Guo et al., 2015; Lenover Moyer et al., 2025; Zhang et al., 2025). Its pathogenesis might also involve the gut-brain axis, visceral hypersensitivity, altered intestinal motility, and microbiome disruption (Simrén et al., 2013). College students, a young demographic facing significant academic and personal pressures alongside irregular routines, exhibit a relatively high overall risk of developing IBS (Alzahrani et al., 2023; Morariu et al., 2023). Highly prevalent and prone to recurrence, IBS significantly impairs patients’ quality of life, disrupting daily activities, work, and social functioning, while imposing a substantial burden on healthcare systems (Gralnek et al., 2000; Huang et al., 2023). In-depth research into IBS is crucial to uncover its underlying mechanisms, thereby alleviating college students’ physical and psychological distress, reducing socioeconomic burdens, and promoting efficient medical resource utilization. Therefore, studying IBS among college students is of great significance.

Anxiety and depression, representing poor mental health, are significant IBS risk factors. Anxiety involves a sense of fear arising from perceived threats or pressure (Dean, 2016). Depression is a negative emotional state characterized by persistent low mood, anhedonia (lack of pleasure), loss of interest, guilt, and low self-evaluation (Malhi and Mann, 2018; Diao et al., 2026). Research has indicated that about 21.48% of Chinese college students may suffer from depressive symptoms (Luo et al., 2025), with anxiety and depressive symptoms remaining common among Chinese college students (Chen et al., 2024). Consequently, anxiety and depression are increasingly common among teenagers and college students, highlighting the significant role of mental health in IBS occurrence and treatment. The relationship between IBS and psychological factors is widely recognized as involving the brain-gut axis (Eijsbouts et al., 2021). Studies show psychological stress can be alleviated through mind–body interactions, effectively assisting IBS treatment (D'Silva et al., 2023). This regulation affects the “brain-gut axis” to alleviate IBS symptoms and comorbid anxiety and depression (Guo et al., 2021). Therefore, intervening in psychological factors is highly significant for IBS treatment.

Physical activity refers to bodily movement that consumes energy through skeletal muscle contraction (Westerterp, 2013). Physical activity is crucial for maintaining good health. Previous studies link insufficient physical activity to various diseases, including obesity, cardiovascular diseases, diabetes, anxiety, depression, and gastrointestinal disorders like IBS (Elagizi et al., 2020; Ho et al., 2024; Oppert et al., 2023; Perry et al., 2023; Singh et al., 2023). Research by (Chen et al., 2024) on sedentary behavior and gastrointestinal diseases identified insufficient physical activity as an important risk factor. Regular physical activity (RPA) involves planning and regulating exercise intensity and frequency to achieve effective goals (Molina-Cantero et al., 2021). According to the World Health Organization (WHO), about one-third of adults worldwide lack sufficient physical activity, with prevalence 5 percentage points higher in women than men (Strain et al., 2024). College students, spending long hours sitting in classrooms and using electronic devices, exhibit high levels of sedentary behavior (Alkatan et al., 2021). Consequently, RPA participation rates among college students are relatively low. Research indicates RPA benefits the nervous system, positively promoting emotional, cognitive, motor skill, and coordination development (Alkatan et al., 2021). Furthermore, RPA is an effective intervention for improving mental health and quality of life (Donnelly et al., 2024).

However, current research on the association between RPA and IBS is limited. Previous studies confirm that poor mental health is an important risk factor for IBS, and RPA helps relieve anxiety and depression (Kutschke et al., 2022; Mahindru et al., 2023). Yet, whether RPA is associated with IBS-related symptom severity through improvements in anxiety and depressive symptoms remains unclear (Hu et al., 2023; Shrestha et al., 2022; Tully et al., 2020). To address this gap, a cross-sectional survey was conducted among 872 students from three universities in Shandong Province, China. Questionnaires covered four dimensions: general condition, RPA status, anxiety/depression assessment scales, and IBS-related symptom severity. The associations between RPA and IBS-related symptom severity were examined, and the mediating roles of depressive symptoms were subsequently evaluated.

## 2 Methods

### 2.1 Participant recruitment

The present study was conducted at three institutions of higher education in Shandong province, China: Shandong First Medical University, Shandong University of Traditional Chinese Medicine, and Heze Medical College. A stratified cluster sampling method was employed. Specifically, 1–3 classes were randomly selected from each grade within each institution, and all students within these selected classes comprised the research subjects. Questionnaire data were collected via on-site surveys, yielding a total of 1,016 completed questionnaires. Eligibility criteria and exclusion criteria are detailed in Supplementary material. Following the application of these criteria and the removal of invalid or incomplete questionnaires, a total of 872 questionnaires were included in the final analysis, representing an effective recovery rate of 85.8%.

### 2.2 Questionnaires

#### 2.2.1 General situation questionnaire

The questionnaire incorporated standard demographic survey items to record respondents’ characteristics, including gender, age, height, and weight. Building on this foundation, an investigation of personal habits: particularly smoking, drinking, and dietary patterns (specifically weekly consumption frequency of chili peppers, dairy products, sugary drinks, and fruits) was added (Alhammadi et al., 2023). All questions employed a closed-ended format, allowing respondents to select the closest matching option.

#### 2.2.2 Physical activity survey form

Physical activity was assessed using the physical activity rating scale (PARS-3) to document respondents’ weekly physical activity patterns, including frequency, total duration, intensity, and other relevant metrics (Yuan and You, 2022). RPA was defined as meeting all of the following criteria (Wang et al., 2021):

- Participation in physical activity ≥ 3 times/week;
- Each session lasting ≥ 30 min;
- Engagement in ≥ 1 moderate- or high-intensity activity.

#### 2.2.3 Self-rating depression scale (SDS)

The SDS was used to assess depressive symptoms using a bidirectional scoring system (positive and negative items) (Zung, 1965; He et al., 2025). Each item is rated on a 1–4 point scale. Following standard scoring procedures, raw scores are multiplied by 1.25 and rounded to yield a total score categorized into:

- Non-depression (< 50 points);
- Mild depression (50–59 points);
- Moderate depression (60–69 points);
- Severe depression (≥ 70 points).

The SDS demonstrated good internal consistency (Cronbach’s *α* = 0.843).

#### 2.2.4 Self-rating anxiety scale (SAS)

The SAS was used to assess anxiety symptoms using a 20-item structure with 1–4 point scoring (Zung, 1971). Total raw scores were multiplied by 1.25 and rounded to the nearest integer to obtain standardized scores. These scores were categorized as follows:

- Non-anxiety (< 50 points);
- Mild anxiety (50–59 points);
- Moderate anxiety (60–69 points);
- Severe anxiety (≥ 70 points).

The SAS demonstrated good reliability (Cronbach’s *α* = 0.822).

#### 2.2.5 Assessment of IBS status and IBS-related symptom severity

IBS status was assessed according to the Rome IV symptom-based criteria (Bai et al., 2017). However, the IBS symptom severity scale (IBS-SSS) was used as a continuous measure of IBS symptom severity across the whole study population rather than as a diagnostic tool (Francis et al., 1997). The scale comprises five items assessing abdominal pain severity, abdominal pain frequency, abdominal distension severity, dissatisfaction with bowel habits, and interference with daily life. Higher scores in each domain indicate greater symptom severity. Total scores classify IBS-related symptom severity into four grades:

- No or minimal symptoms (<75 points)
- Mild symptom (75–175 points);
- Moderate symptom (175–300 points);
- Severe symptom (> 300 points).

The scale demonstrated good reliability (Cronbach’s *α* = 0.818).

#### 2.2.6 Gastrointestinal symptom rating scale (GSRS)

The GSRS assesses gastrointestinal symptom severity across five dimensions: abdominal pain, reflux, diarrhea, dyspepsia, and constipation (Kulich et al., 2008). Each dimension is scored on a 1–7 point scale, where 1 indicates absence of symptoms. The sum of subscale scores yields the total score, with higher scores reflecting greater symptom severity. The GSRS demonstrated excellent internal consistency (Cronbach’s *α* = 0.932).

### 2.3 Statistical analysis

Statistical analyses were performed using IBM SPSS 26.0 and R 4.2.1. Baseline characteristics of the participants were described using frequencies and proportions. Categorical variables were compared between groups using chi-square tests. Normality was assessed using the Kolmogorov–Smirnov test, and all variables were non-normally distributed. Comparisons of IBS-SSS and GSRS scores were conducted using the Mann–Whitney U test and the Kruskal-Wallis H test. Multiple linear regression analyses were performed to examine the associations of RPA status, SDS scores, and SAS scores with IBS-SSS and GSRS scores. Potential covariates were selected based on their associations with IBS-related symptom severity in group comparisons. The assumptions of linear regression, including linearity, approximate normality of residuals, and influential observations, were assessed using residual diagnostic plots, with diagnostic results presented in Supplementary Figures S1, S2. Exploratory mediation analyses were performed in R using the mediation package (version 4.5.1). Simple mediation models were fitted separately for IBS-SSS and GSRS scores. RPA was specified as the exposure variable, and SDS scores were evaluated as the mediator. RPA status was coded as 0 for non-RPA and 1 for RPA. The mediator and outcome models were fitted using multiple linear regression. Non-parametric bootstrap resampling with 5,000 simulations was used to estimate 95% confidence intervals. All statistical tests were two-sided, and a significance level of *α* = 0.05 was applied.

## 3 Results

### 3.1 General characteristics of the study population

The present study comprised 872 participants, including 264 males (30.3%) and 608 females (69.7%) (Table 1). The median age was 21 years. Participants were stratified into age groups of < 20 years (9.6%), 20–25 years (86.4%), and ≥ 25 years (4.0%). Educational backgrounds consisted of college students (53.1%), university students (41.3%), and postgraduates (5.6%). Median BMI measured 21.0 kg/m^2^ (IQR: 19.6–23.0), distributed across underweight (13.3%), normal weight (62.7%), overweight (15.7%), and obese (8.3%) categories. Residential distribution showed 67.2% rural and 32.8% urban dwellers. Sleep duration analysis indicated that 0.6% slept < 4 h/day, 8.7% slept 4–6 h, 77.2% slept 6–8 h, and 13.5% slept ≥ 8 h. Passive smoking exposure occurred less than once weekly among 54.4% of participants. Weekly passive smoking frequencies included 1–3 exposures for 37.3% of subjects, 4–6 exposures for 5.7%, and ≥ 7 exposures for 2.6%. Regarding alcohol consumption, 79.3% never drank alcohol, while 19.2% consumed alcohol less than twice weekly, and 1.5% drank ≥ 2 times weekly. Spicy food consumption patterns revealed that 11.2% never consumed spicy foods. Weekly spicy food intake included < 2 times for 41.1%, 3–5 times for 35.7%, and ≥ 6 times for 12.0% of participants. Yogurt consumption showed 16.7% never consumed yogurt, with 59.6% eating yogurt < 2 times weekly. Higher yogurt intake involved 3–5 times weekly (20.8%) and ≥ 6 times weekly (2.9%). Takeaway meal consumption indicated that 31.3% never ordered takeaway. Weekly takeaway consumption comprised < 2 orders for 51.7%, 3–5 orders for 11.7%, and ≥ 6 orders for 5.3%. Sugar-sweetened beverage intake demonstrated that 15.0% never consumed these drinks. Weekly consumption patterns included < 2 times (58.0%), 3–5 times (21.9%), and ≥ 6 times (5.1%). Fruit consumption analysis found that 5.2% never ate fruit. Weekly fruit intake involved < 2 times (40.2%), 3–5 times (41.4%), and ≥ 6 times (13.2%) consumption frequencies.

**Table 1:** Baseline characteristics of the study population (*n* = 872).

Variables	RPA	Non-RPA	*p*
	*n* (%)	*n* (%)	
Gender			
Male	74 (28.0)	190 (72.0)	< 0.001**
Female	80 (13.2)	528 (86.8)	
Age (years)			
< 20	12 (14.3)	72 (85.7)	0.330
20–25	133 (17.7)	620 (82.3)	
≥ 25	9 (25.7)	26 (74.3)	
Educational level			
Junior college student	66 (14.3)	397 (85.7)	0.004**
Undergraduate student	73 (20.3)	287 (79.7)	
Postgraduate student	15 (30.6)	34 (69.4)	
BMI (kg/m^2^)			
< 18.5	12 (10.3)	104 (89.7)	0.001**
18.5–24.0	89 (16.3)	458 (83.7)	
24.0–28.0	30 (21.9)	107 (78.1)	
≥ 28.0	23 (31.9)	49 (68.1)	
Domicile			
Rural	104 (17.7)	482 (82.3)	0.923
Urban	50 (17.5)	236 (82.5)	
Amount of sleep (hours)			
< 4	1 (20.0)	4 (80.0)	0.586
4–6	10 (13.2)	66 (86.8)	
6–8	125 (18.6)	548 (81.4)	
≥ 8	18 (15.3)	100 (84.7)	
Smoking habit			
No	146 (17.4)	695 (82.6)	0.226
Yes^a^	8 (25.8)	23 (74.2)	
Passive smoking^b^			
< 1 time a week	88 (18.6)	386 (81.4)	0.663
1–3 times a week	51 (15.7)	274 (84.3)	
4–6 times a week	10 (20.0)	40 (80.0)	
≥ 7 times a week	5 (21.7)	18 (78.3)	
Alcohol consumption			
Never	107 (15.5)	585(84.5)	0.003**
< 2 times a week	43 (25.7)	124(74.3)	
≥ 2 times a week	4 (30.8)	9(69.2)	
Spicy food consumption			
Never	18 (18.4)	80 (81.6)	0.599
< 2 times a week	60 (16.8)	298 (83.2)	
3–5 times a week	61 (19.6)	250 (80.4)	
≥ 6 times a week	15 (14.3)	90 (85.7)	
Yoghurt consumption			
Never	34 (23.3)	112 (76.7)	0.281
< 2 times a week	86 (16.5)	434 (83.5)	
3–5 times a week	30 (16.6)	151 (83.4)	
≥ 6 times a week	4 (16.0)	21 (84.0)	
Takeaway consumption			
Never	52 (19.0)	221 (81.0)	0.232
< 2 times a week	70 (15.5)	381 (84.5)	
3–5 times a week	20 (19.6)	82 (80.4)	
≥ 6 times a week	12 (26.1)	34 (73.9)	
Sugar-sweetened beverage consumption			
Never	34 (26.0)	97 (74.0)	0.044*
< 2 times a week	85 (16.8)	421 (83.2)	
3–5 times a week	27 (14.1)	164 (85.9)	
≥ 6 times a week	8 (18.2)	36 (81.8)	
Fruit consumption			
Never	11 (24.4)	34 (75.6)	0.012*
< 2 times a week	48 (13.7)	303 (86.3)	
3–5 times a week	65 (18.0)	296 (82.0)	
≥ 6 times a week	30 (26.1)	85 (73.9)	

a Current smokers and past smokers were included.

b Exposure to smoke caused by smokers for more than 15 min is counted as a single exposure.

RPA, regular physical activity; BMI, body mass index; *p < 0.05; **p < 0.01.

### 3.2 Participation of physical activity

In the studied population, statistical analysis revealed an RPA participation rate of 17.7%. Physical activity frequency was categorized into five weekly groups: 246 participants (28.2%) exercised less than once weekly, indicating most lacked regular physical activity habits; another 246 (28.2%) engaged in activity 1–2 times/week, suggesting widespread low-frequency participation; 228 (26.1%) participated 3–4 times weekly; 128 (14.7%) engaged 5–7 times/week, representing a small high-frequency cohort; while only 24 (2.8%) exercised ≥ 8 times weekly (Table 2). Regarding session duration, 264 participants (30.3%) had sessions lasting < 15 min, and 354 (40.6%) exercised 15–30 min per session - indicating insufficient duration for most. Longer durations included 169 (19.4%) at 30–60 min, 64 (7.3%) at 60–90 min, and 21 (2.4%) at ≥ 90 min, demonstrating a small but consistent long-duration group. Post-activity symptoms analysis showed: 169 participants (19.4%) exhibited minimal breathing/heartbeat changes without sweating (LCBH); 489 (56.1%) displayed slightly accelerated breathing/heartbeat with mild sweating (BHSUS); 172 (19.7%) demonstrated fast breathing/heartbeat with moderate sweating (FBH); and 42 (4.8%) experienced rapid breathing/heartbeat with profuse sweating (RBH).

**Table 2:** IBS-SSS and GSRS scores in the study population (*n* = 872).

Variables	*n*	IBS-SSS score			GSRS score		
		Mean ± SD	Median	*p*	Mean ± SD	Median	*p*
Gender							
Male	264	73.5 ± 75.8	50.0	0.002^a^	25.8 ± 13.5	21.0	<0.001^a^
Female	608	82.5 ± 61.5	75.0		28.3 ± 12.4	25.0	
Age (years)							
< 20	84	78.9 ± 71.1	75.0	0.218^b^	27.9 ± 13.0	24.0	0.631^b^
20–25	753	80.6 ± 65.9	75.0		27.6 ± 12.7	24.0	
≥ 25	35	62.9 ± 60.7	50.0		25.9 ± 13.1	21.0	
Educational level							
Junior college student	463	74.8 ± 68.1	50.0	0.005^b^	27.1 ± 13.1	23.0	0.093^b^
Undergraduate student	360	87.1 ± 63.2	100.0		28.0 ± 12.2	25.0	
Postgraduate student	49	73.0 ± 65.9	75.0		28.6 ± 13.9	25.0	
BMI (kg/m^2^)							
< 18.5	116	67.2 ± 60.7	50.0	0.076^b^	25.5 ± 10.2	22.0	0.304^b^
18.5–24.0	547	82.8 ± 66.3	75.0		27.9 ± 13.1	24.0	
24.0–28.0	137	82.3 ± 66.6	75.0		28.7 ± 13.8	25.0	
≥ 28.0	72	72.2 ± 71.8	50.0		25.7 ± 10.9	22.0	
Domicile							
Rural	586	79.4 ± 67.5	75.0	0.622^a^	27.7 ± 12.9	24.0	0.796^a^
Urban	286	80.6 ± 63.6	75.0		27.3 ± 12.4	24.0	
Amount of sleep (hours)							
< 4	5	100.0 ± 106.1	100.0	0.038^b^	33.2 ± 16.7	43.0	<0.001^b^
4–6	76	102.6 ± 73.3	100.0		35.1 ± 16.2	28.5	
6–8	673	77.7 ± 64.8	75.0		26.7 ± 11.6	24.0	
≥ 8	118	75.8 ± 65.4	75.0		27.3 ± 14.6	22.0	
Smoking habit							
No	841	80.1 ± 65.9	75.0	0.195^a^	27.6 ± 12.8	24.0	0.479^a^
Yes^c^	31	69.4 ± 74.9	50.0		26.3 ± 12.0	22.0	
Passive smoking^d^							
< 1 time a week	474	71.6 ± 66.2	50.0	0.072^b^	26.0 ± 11.9	22.0	0.350^b^
1–3 times a week	325	87.8 ± 62.6	100.0		28.4 ± 12.7	25.0	
4–6 times a week	50	100.0 ± 64.1	100.0		33.4 ± 15.6	30.0	
≥ 7 times a week	23	90.2 ± 95.3	50.0		33.2 ± 16.9	28.0	
Alcohol consumption							
Never	692	78.3 ± 64.0	75.0	0.061^b^	27.0 ± 12.2	24.0	0.070^b^
< 2 times a week	167	81.4 ± 71.2	75.0		28.7 ± 12.8	25.0	
≥ 2 times a week	13	136.5 ± 91.6	150.0		42.4 ± 26.1	37.0	
Spicy food consumption							
Never	98	77.8 ± 79.3	50.0	0.392^b^	25.3 ± 13.2	20.5	0.020^b^
< 2 times a week	358	77.0 ± 64.2	75.0		27.0 ± 11.7	24.0	
3–5 times a week	311	81.8 ± 63.8	75.0		28.5 ± 13.0	25.0	
≥ 6 times a week	105	85.0 ± 67.1	75.0		28.6 ± 14.7	24.0	
Yoghurt consumption							
Never	146	78.9 ± 70.6	75.0	0.121^b^	27.1 ± 12.5	22.5	0.398^b^
< 2 times a week	520	78.6 ± 65.4	75.0		27.1 ± 12.1	24.0	
3–5 times a week	181	79.7 ± 64.5	75.0		28.6 ± 14.1	25.0	
≥ 6 times a week	25	109.0 ± 65.3	125.0		32.6 ± 16.5	28.0	
Takeaway consumption							
Never	273	71.4 ± 63.8	50.0	<0.001^b^	26.5 ± 12.2	23.0	0.049^b^
< 2 times a week	451	78.9 ± 65.1	75.0		27.5 ± 12.7	24.0	
3–5 times a week	102	89.0 ± 66.4	100.0		30.1 ± 14.0	28.0	
≥ 6 times a week	46	117.4 ± 76.9	100.0		28.1 ± 12.7	26.0	
Sugar-sweetened beverage consumption							
Never	131	71.0 ± 68.9	50.0	0.028^b^	25.3 ± 12.1	21.0	<0.001^b^
< 2 times a week	506	78.0 ± 63.9	75.0		26.7 ± 11.8	24.0	
3–5 times a week	191	84.8 ± 64.4	100.0		30.1 ± 13.4	27.0	
≥ 6 times a week	44	104.0 ± 84.6	100.0		33.3 ± 18.0	26.5	
Fruit consumption							
Never	45	88.9 ± 85.2	75.0	0.254^b^	28.0 ± 14.8	24.0	0.124^b^
< 2 times a week	351	78.2 ± 62.2	75.0		26.7 ± 11.2	24.0	
3–5 times a week	361	83.1 ± 66.5	75.0		28.8 ± 13.8	25.0	
≥ 6 times a week	115	70.4 ± 68.4	50.0		26.1 ± 12.7	22.0	
Frequency of physical activity							
< 1 time a week	246	92.6 ± 68.1	100.0	<0.001^b^	30.2 ± 14.2	26.0	0.006^b^
1–2 times a week	246	79.0 ± 64.7	75.0		26.7 ± 12.1	24.0	
3–4 times a week	228	78.3 ± 66.5	75.0		26.8 ± 11.6	24.0	
5–7 times a week	128	64.3 ± 60.2	50.0		25.2 ± 10.5	23.0	
≥ 8 times a week	24	53.1 ± 67.7	25.0		29.0 ± 19.3	21.0	
Duration of each physical activity (minutes)							
< 15	264	89.2 ± 68.0	100.0	<0.001^b^	29.6 ± 13.8	26.0	0.008^b^
15–30	354	82.3 ± 64.3	87.5		27.4 ± 12.5	24.0	
30–60	169	65.8 ± 64.8	50.0		25.2 ± 11.3	22.0	
60–90	64	71.9 ± 64.2	50.0		27.0 ± 12.1	24.0	
≥ 90	21	54.8 ± 72.7	0.0		24.9 ± 13.4	17.0	
Symptoms after physical activity							
LCBH	169	85.9 ± 72.0	75.0	0.659^b^	28.6 ± 14.3	24.0	0.258^b^
BHSUS	489	77.0 ± 64.0	75.0		27.1 ± 12.1	24.0	
FBH	172	79.7 ± 62.2	75.0		26.8 ± 11.8	24.5	
RBH	42	86.9 ± 81.8	75.0		32.1 ± 16.5	27.5	

a Mann–Whitney U test.

b Kruskal-Wallis H test.

c Current smokers and past smokers were included.

d Exposure to smoke caused by smokers for more than 15 min is counted as a single exposure.

GSRS, gastrointestinal symptom rating scale; IBS-SSS, irritable bowel syndrome symptom severity scale; SD, standard deviation; BMI, body mass index; LCBH, little change in breathing and heartbeat, usually no sweating; BHSUS, breathing and heartbeat sped up slightly, sweating slightly; FBH, fast breathing and heartbeat, sweating more; RBH, rapid breathing and heartbeat, sweating profusely.

Furthermore, the study population was stratified by sociodemographic characteristics for group comparisons (Table 1). Results showed men participated in RPA at a significantly higher rate than women (28.0% vs. 13.2%, *p* < 0.001), a difference potentially linked to sociocultural factors and traditional gender perceptions. Significant variations in RPA participation emerged across educational levels (*p* = 0.004). Postgraduate students showed higher participation than undergraduates, who in turn showed higher participation than junior college students. RPA participation also differed significantly across BMI groups (*p* = 0.001), with higher BMI associated with greater participation. Significant differences were further observed based on weekly alcohol consumption frequency (*p* = 0.003) and weekly sugar-sweetened beverage consumption (*p* = 0.044). Fruit consumption similarly correlated with RPA participation (*p* = 0.012). However, no significant associations were found between RPA participation and age, domicile, sleep duration, smoking, or dietary factors (spicy food consumption, yogurt intake, or takeaway frequency) (all *p* > 0.05).

### 3.3 IBS-related symptom severity

Analysis of the integrated survey data revealed a population median IBS-SSS score of 75.0 (IQR: 25.0–125.0) and a median GSRS score of 24.0 (IQR: 18.0–33.0). Among the 872 participants, 20 (2.3%) met the Rome IV symptom-based criteria for IBS. The IBS-SSS was subsequently used to evaluate IBS symptom severity in the entire study population. Based on IBS-SSS scores, 389 participants (44.6%) had no or minimal symptoms, 402 (46.1%) had mild symptoms, 77 (8.8%) had moderate symptoms, and only 4 (0.5%) had severe symptoms. Stratification by sociodemographic characteristics showed significant group differences (Table 2 and Supplementary Table S1). Among males (*n* = 264), 141 (53.4%) had no or minimal, 92 (34.8%) had mild, 28 (10.6%) moderate, and 3 (1.1%) severe symptoms. Among females (*n* = 608), 248 (40.8%) had no or minimal, 310 (51.0%) had mild, 49 (8.1%) moderate, and 1 (0.2%) severe symptoms, with females exhibiting higher overall symptom scores. Gender significantly impacted both IBS-SSS (*p* = 0.002) and GSRS scores (*p* < 0.001). Significant differences in IBS-SSS scores were also observed across educational levels (*p* = 0.005): no/minimal symptoms were observed in 50.5% of junior college students, 36.7% of undergraduates, and 46.9% of postgraduates, while mild symptoms were observed in 40.8, 53.3, and 42.9%, respectively., indicating higher IBS-SSS among undergraduates compared to junior college students. Sleep duration was significantly associated with IBS-SSS (*p* = 0.038) and GSRS scores (*p* < 0.001), showing a trend where shorter sleep correlated with higher symptom severity. Specifically, participants sleeping < 4 h showed 40.0% no/minimal, 40% mild and 20% moderate symptoms; those sleeping 4–6 h showed 32.9% no/minimal, 53.9% mild, 10.5% moderate, and 2.6% severe; the majority sleeping 6–8 h showed 45.3% no/minimal, 46.1% mild, 8.3% moderate, and 0.3% severe; and those sleeping ≥ 8 h showed 48.3% no/minimal, 41.5% mild and 10.2% moderate symptoms. Dietary analysis showed the frequency of spicy food consumption significantly affected GSRS severity (*p* = 0.020), takeaway consumption affected both IBS-SSS (*p* < 0.001) and GSRS scores (*p* = 0.049), and sugary beverage consumption affected both IBS-SSS (*p* = 0.028) and GSRS scores (*p* < 0.001). No significant correlations were found between IBS-SSS/GSRS score and age, BMI, domicile, smoking, alcohol habits, yogurt consumption, or fruit consumption (all *p* > 0.05).

### 3.4 Occurrence of depressive and anxiety symptoms

Regarding anxiety and depression, the surveyed population was assessed using the SAS and SDS (Supplementary Table S2). Analysis of valid SDS data revealed that 413 individuals (47.4%) exhibited no depressive symptoms, while 325 (37.3%) experienced mild depressive symptoms, 119 (13.6%) had moderate depressive symptoms, and 15 (1.7%) were classified as having severe depressive symptoms. Analysis of the SAS data indicated that 588 individuals (67.4%) had no anxiety symptoms, 227 (26.0%) showed mild anxiety symptoms, 46 (5.3%) presented with moderate anxiety symptoms, and 11 (1.3%) had severe anxiety symptoms.

As shown in Figure 1A, the proportion of individuals without depressive symptoms was higher in the RPA group than in the non-RPA group (57.1% vs. 45.3%), while the proportion with severe depressive symptoms was lower (0.7% vs. 1.9%) (*p* < 0.05). Figure 1B demonstrated a significant difference in depressive symptom levels between groups based on SDS scores, with the RPA group exhibiting significantly lower scores than the non-RPA group, indicating reduced depressive symptom severity (*p* < 0.01). Figure 1C showed the RPA group had a higher proportion of individuals without anxiety symptoms compared to the non-RPA group (73.4% vs. 66.2%) and a higher proportion with severe anxiety symptoms; however, no significant difference was observed in anxiety symptom severity (*p* > 0.05). Figure 1D confirmed no statistically significant difference in SAS scores between groups (*p* > 0.05).

**Figure 1:**
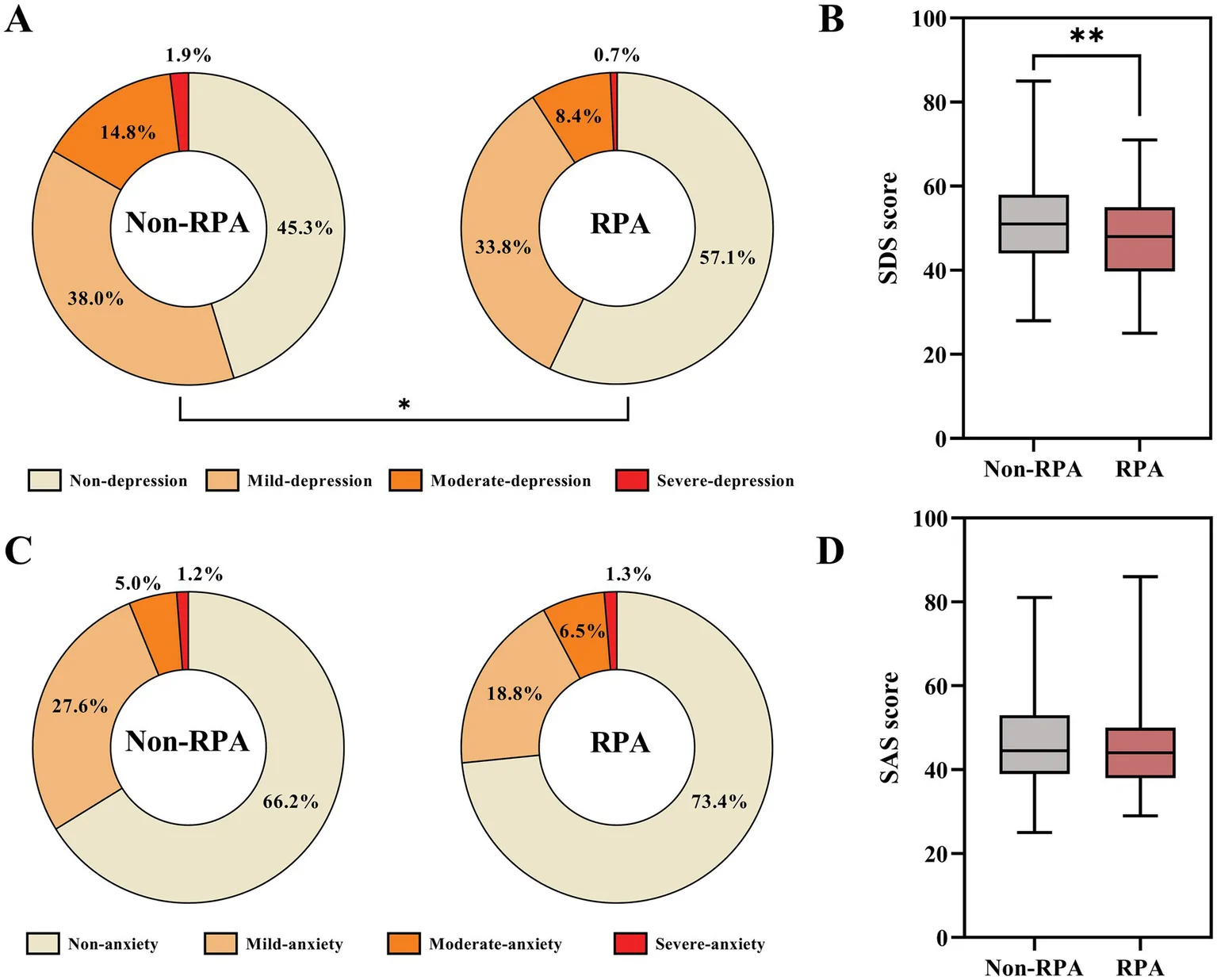
Depressive and anxiety symptoms in the non-RPA and RPA groups (*n* = 872). **(A)** Distribution of depressive symptoms in non-RPA and RPA groups based on SDS categories; **(B)** Boxplot of SDS scores between non-RPA and RPA groups; **(C)** Distribution of anxiety symptoms in non-RPA and RPA groups based on SAS categories; **(D)** Boxplot of SAS scores between non-RPA and RPA groups; RPA, Regular physical activity; SDS, Self-rating depression scale; SAS, Self-rating anxiety scale; *p < 0.05; **p < 0.01.

### 3.5 Association between RPA and IBS-related symptom severity

The distributions of IBS-SSS and GSRS scores, as well as IBS-related symptom severity, across participant characteristics are presented in Table 2 and Supplementary Table S1. In terms of physical activity frequency, among those exercising less than once per week, 36.2% had no/minimal symptoms, 51.2% had mild symptoms and 11.8% had moderate symptoms, while 0.8% had severe symptoms; among those active 1–2 times per week, 43.5% had no/minimal symptoms, 48.4% presented with mild, 7.3% with moderate, and 0.8% with severe symptoms; for the 3–4 times per week group, no/minimal, mild, and moderate symptoms accounted for 47.8, 43.0, and 9.2%, respectively; and among those active 5–7 times or ≥ 8 times per week, no/minimal, mild, and moderate symptoms accounted for 53.1%/41.4%/5.5% and 66.7%/25.0%/8.3%, respectively, indicating that lower activity frequency was associated with higher IBS-SSS scores (*p* < 0.001) and higher GSRS scores (*p* = 0.006), as well as greater gastrointestinal symptom burden. Regarding the duration per activity session, among those with sessions < 15 min, no/minimal, mild, moderate, and severe symptoms accounted for 37.9, 50.4, 11.4, and 0.4%, respectively; in the 15–30 min group, the proportions were 42.1, 49.4, 7.9, and 0.6%; for the 30–60 min group, they were 55.0, 38.5, 5.9, and 0.6%; and within both the 60–90 min and ≥ 90 min groups, no/minimal, mild, and moderate symptoms accounted for 53.1%/35.9%/10.9% and 61.9%/28.6%/9.5%, respectively, showing that longer activity duration was significantly associated with lower IBS-SSS (*p* < 0.001) and GSRS scores (*p* = 0.008), a trend consistent with real-world observations. No significant correlation was found between symptoms following physical activity and IBS-SSS or GSRS.

As shown in Figure 2, both IBS-SSS and GSRS scores were significantly lower in the RPA group compared to the non-RPA group (all *p* < 0.01, Figures 2A,B). Multiple linear regression further revealed a significant negative association between RPA participation and IBS-SSS scores [*β* = −28.847, 95% *CI*: (−40.235, −17.459), *p* < 0.01]. After adjusting for confounders including gender, educational level, sleep duration, and dietary habits (frequency of takeaway and sugar-sweetened beverage consumption), this negative association remained statistically significant [*β* = −28.665, 95% *CI*: (−40.129, −17.201), *p* < 0.01]. Similarly, RPA was also negatively associated with GSRS scores in both unadjusted [*β* = −4.359, 95% *CI*: (−6.564, −2.154), *p* < 0.01] and adjusted models that accounted for gender, sleep duration, and dietary factors (spicy food, takeaway, and sugar-sweetened beverage consumption) [*β* = −3.538, 95% *CI*: (−5.740, −1.335), *p* < 0.01].

**Figure 2:**
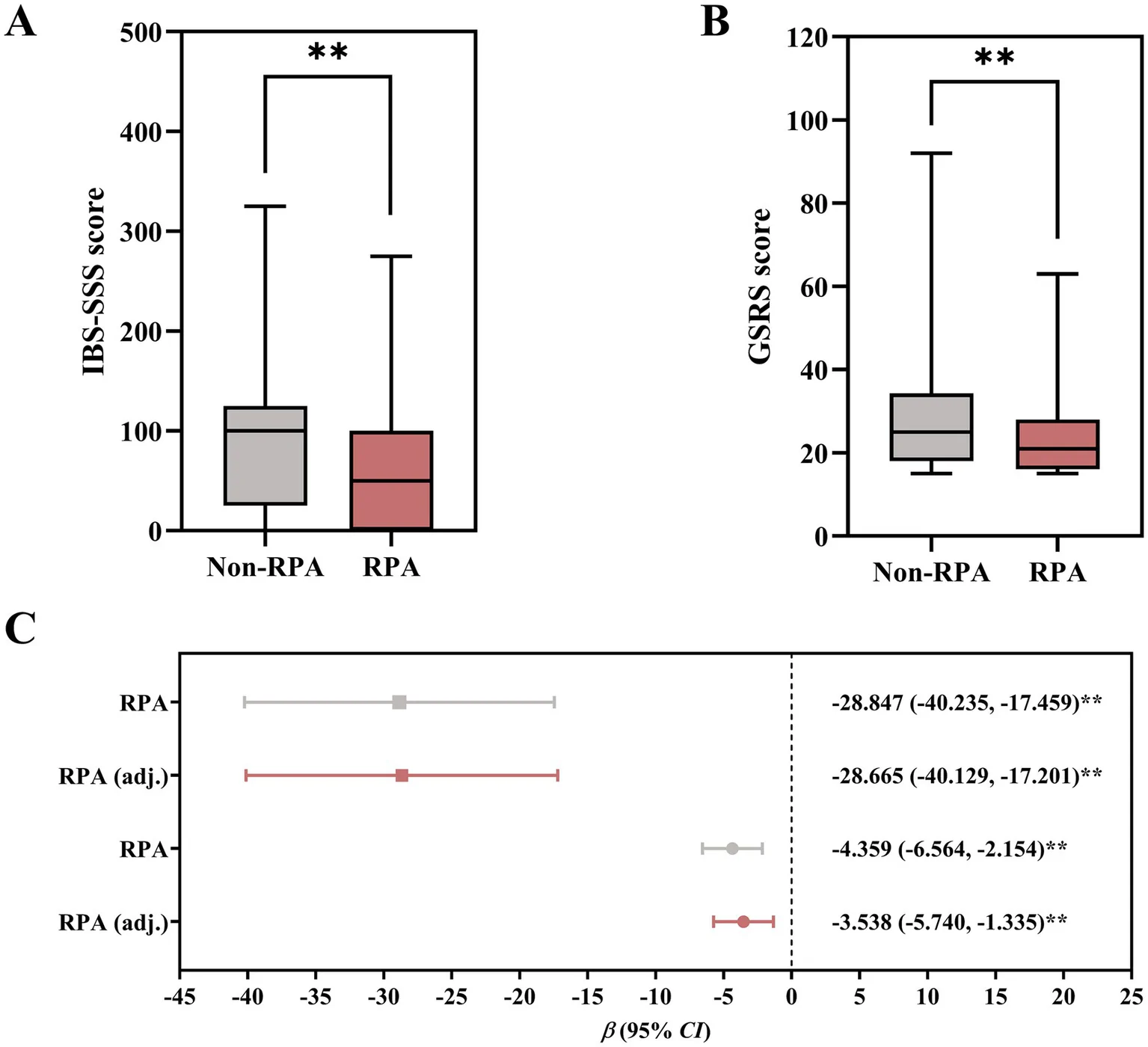
IBS-SSS and GSRS scores in the RPA and non-RPA groups and regression estimates of their associations with RPA (*n* = 872). **(A)** IBS-SSS scores in the non-RPA and RPA groups; **(B)** GSRS scores in the non-RPA and RPA groups; **(C)** Unadjusted and adjusted *β* with 95% CIs for the associations between RPA and IBS-SSS (upper two estimates) and GSRS (lower two estimates) scores. For IBS-SSS, adjusted models controlled for gender, educational level, sleep duration, and dietary habits (frequency of takeaway and sugar-sweetened beverage consumption). For GSRS, adjusted models controlled for gender, sleep duration, and dietary factors (spicy food, takeaway, and sugar-sweetened beverage consumption). GSRS, Gastrointestinal symptom rating scale; IBS-SSS, Irritable bowel syndrome symptom severity scale; RPA, Regular physical activity; *β*, Regression coefficients; CI, Confidence interval; **p < 0.01.

### 3.6 Association between depressive and anxiety symptoms and IBS-related symptom severity

As shown in Supplementary Table S3, both anxiety and depressive symptoms were significantly associated with greater gastrointestinal symptom severity. For IBS-SSS scores, higher SAS scores were positively associated with IBS-related symptom severity in both crude and adjusted models. After adjustment for potential confounders, each unit increase in SAS score was associated with a 2.458-point increase in IBS-SSS score [*β* = 2.458, 95% *CI*: (2.026, 2.890), *p* < 0.001]. Similarly, SDS scores remained significantly associated with IBS-SSS scores after adjustment [*β* = 1.745, 95% *CI*: (1.318, 2.172), *p* < 0.001]. For GSRS scores, SAS scores were positively associated with GSRS scores in the adjusted model [*β* = 0.549, 95% *CI*: (0.468, 0.629), *p* < 0.001]. Likewise, higher SDS scores were associated with increased GSRS scores [*β* = 0.380, 95% *CI*: (0.299, 0.461), *p* < 0.001]. These findings indicate that depressive and anxiety symptom severity were independently associated with greater gastrointestinal symptom burden after accounting for relevant covariates.

### 3.7 Mediating role of depressive symptoms between RPA and IBS-related symptom severity

Mediation analysis was conducted to evaluate the role of depressive symptoms in the association between RPA and IBS-related symptom severity. Mediation models were adjusted using the same covariates as the corresponding multivariable regression models. As shown in Figure 3A, the estimated indirect association between RPA and GSRS scores through SDS scores was −1.224 [95% *CI*: (−1.992, −0.562), *p* < 0.001], with an estimated proportion mediated of 34.36% (*p* < 0.001). Similarly, as shown in Figure 3B, the estimated indirect association between RPA and IBS-SSS scores through SDS scores was −5.659 [95% *CI*: (−9.199, −2.644), *p* < 0.001] and a mediation proportion of 19.43% (*p* < 0.001). These findings suggest that depressive symptoms statistically accounted for part of the observed association between RPA and IBS-related symptom severity.

**Figure 3:**
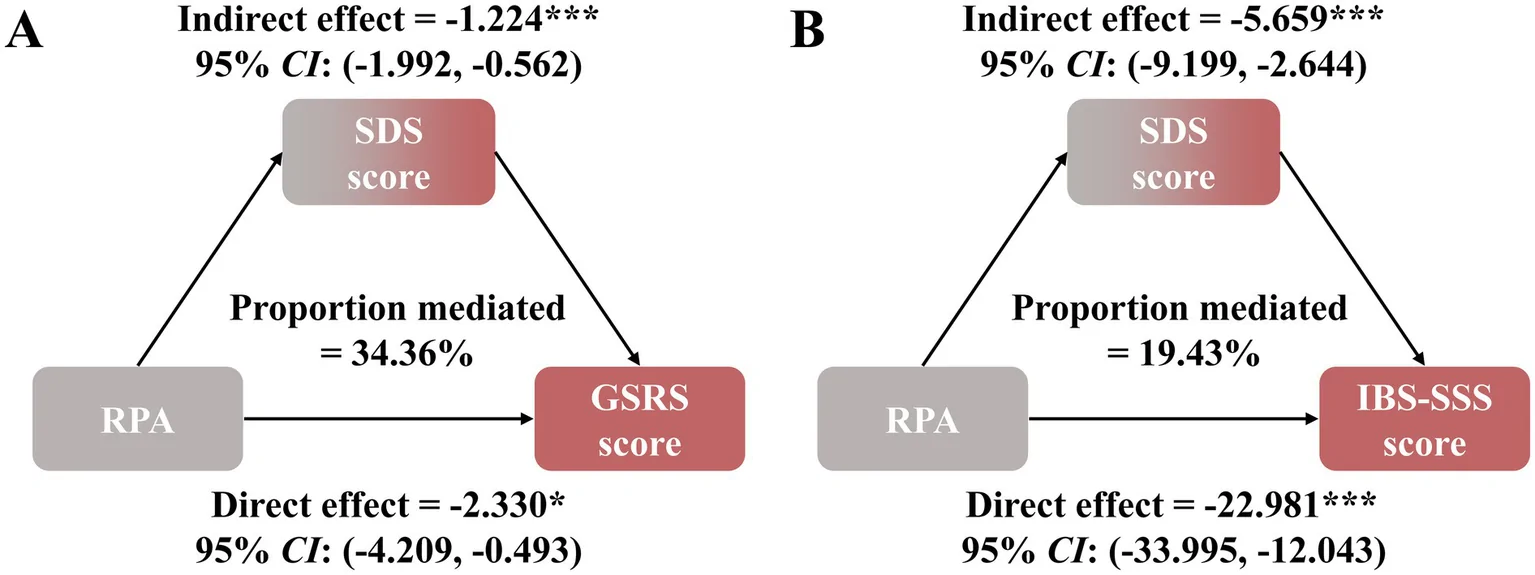
Mediating role of depressive symptoms in the relationship between RPA and IBS-related symptom severity (*n* = 872). **(A)** Mediating role of the SDS score in the association between RPA and the GSRS score; **(B)** Mediating role of the SDS score in the association between RPA and the IBS-SSS score. For IBS-SSS, model controlled for gender, educational level, sleep duration, and dietary habits (frequency of takeaway and sugar-sweetened beverage consumption). For GSRS, model controlled for gender, sleep duration, and dietary factors (spicy food, takeaway, and sugar-sweetened beverage consumption). RPA, Regular physical activity; SDS, Self-rating depression scale; GSRS, Gastrointestinal symptom rating scale; IBS, Irritable bowel syndrome; IBS-SSS, Irritable bowel syndrome symptom severity scale; *p < 0.05; ***p < 0.001.

## 4 Discussion

In this cross-sectional study of Chinese college students, significant associations were observed between RPA, psychological symptoms, and gastrointestinal outcomes. The principal findings indicate that RPA was independently associated with lower IBS-related and reduced gastrointestinal symptom burden, and that depressive symptoms statistically accounted for part of these associations. Together, these findings suggest that psychological factors may represent a potential pathway linking RPA with gastrointestinal health.

As in previous studies, male students were more likely than female students to engage in RPA. This gender difference is prevalent globally and may reflect different cultural attitudes towards exercise (The Lancet Public Health, 2019). We also found that graduate students and those with higher BMI indices exhibited higher rates of RPA participation, which could indicate greater health awareness or motivation among these groups (Kari et al., 2020; Bombak, 2015). With regard to IBS-related symptoms, females generally experienced a greater symptom burden than males, consistent with other studies (Bonetto et al., 2021; Arif et al., 2025). Symptom severity differed across groups defined by several behavioral factors, including sleep duration and the frequency of spicy food, sugar-sweetened beverage and takeaway meal consumption. The differences between spicy food intake groups may be explained by capsaicin-induced activation of transient receptor potential vanilloid 1 (TRPV1), which exacerbates visceral pain and burning sensations in individuals with IBS (Gonlachanvit, 2010). Large quantities of fermentable sugars in sugar-sweetened beverages may enhance colonic fermentation, leading to gas production, bloating, and diarrhea (Wu et al., 2025). Furthermore, takeaway foods often have a high sugar and fat content, and may be subject to inadequate food safety oversight. This can increase the risk of gastrointestinal disturbances and low-grade inflammation, which could exacerbate gastrointestinal symptoms associated with IBS (Jin et al., 2025). These observations suggest that modifiable habits such as sleep hygiene and diet, in addition to exercise, may influence IBS-related symptom severity, which is consistent with prior research (Ho et al., 2024).

College students who engaged in RPA had significantly lower IBS-SSS and GSRS scores than their sedentary peers. This finding is consistent with a UK Biobank study which showed that high-intensity exercise reduces IBS incidence (Ho et al., 2024). Furthermore, Mendelian randomization studies indicate that a genetic predisposition to sedentary behavior increases IBS risk, whereas markers for moderate-to-high-intensity exercise predict a lower risk (Chen et al., 2024). The mechanisms underlying the potential beneficial associations between physical activity and IBS-related symptoms remain unclear, but may be related to the following physiological processes. Regular exercise has been shown to promote the production of short-chain fatty acids (SCFAs) and maintain the integrity of the intestinal epithelial barrier (Shin et al., 2023). In addition, SCFAs can modulate immune responses, alleviating low-grade intestinal inflammation associated with the pathogenesis of IBS (Liu et al., 2023). Moderate-intensity physical activity has also been found to enhance vagal tone and parasympathetic nervous system activity (Li et al., 2026; Mahalakshmi et al., 2020), factors critical in regulating gastrointestinal motility, secretory function, and mucosal immunity (Al-Beltagi et al., 2025). Moreover, studies have demonstrated that physical activity can reduce blood cortisol levels (De Nys et al., 2022), and this stress-related hormone may be associated with increased small intestinal permeability and exacerbated gastrointestinal symptoms (Vanuytsel et al., 2014).

Mental health has been identified as a further significant link between RPA and IBS. The findings of this study demonstrated that students who met the criteria for RPA exhibited significantly lower levels of depression compared to their inactive counterparts. Substantial evidence confirms the benefits of exercise for emotional regulation, with physically active individuals demonstrating greater resilience to stress and lower rates of anxiety and depression (Carek et al., 2011; Banyard et al., 2025; Nowacka-Chmielewska et al., 2022). In addition, anxiety and depression symptoms were positively associated with IBS-related symptom severity. Chronic stress and negative emotions exacerbate IBS through neuroimmune and neuroendocrine pathways (Chang, 2011). Moreover, the hypothalamic–pituitary–adrenal (HPA) axis becomes dysregulated in IBS, making patients’ intestines more susceptible to the effects of stressful events (Staudacher et al., 2023). Recent metagenomic evidence in patients with major depressive disorder has also identified associations between gut microbial features, microbial functional pathways, and psychiatric symptom severity, further supporting the potential relevance of microbiota–brain interactions in depressive states (He et al., 2026). Based on the above findings, mediation analysis was employed to explore whether depressive symptoms may partly explain the association between RPA and IBS-related symptom severity. Results indicate that depressive symptoms statistically accounted for part of the association between RPA and IBS-related symptom severity. This finding suggests that RPA may offer a potential therapeutic benefit in the management of IBS-related symptom, possibly by modulating mood. Conversely, anxiety did not show a statistically significant mediating association, which may be indicative of either lower anxiety prevalence or differing severity among the younger study participants. Nevertheless, neuroimaging research in patients with anxious major depressive disorder has linked cognitive dysfunction to altered amygdala functional connectivity, highlighting the neurobiological complexity of co-occurring anxiety and depressive symptoms (Chen et al., 2026). This underscores the notion that mental health is a pivotal co-factor in IBS, with interventions such as exercise, which enhance mood and stress coping abilities, potentially resulting in indirect alleviation of gut symptoms (Li et al., 2024).

A significant innovation of this study is the identification of depressive symptoms as a partial mediator in the relationship between RPA and IBS-related symptom severity. To the best of our knowledge, this is among the first population-based studies suggesting that regular exercise may partially alleviate IBS-related symptom severity by improving mood. This finding is of clinical significance, indicating that the promotion of exercise provides dual benefits for college students, enhancing mental health while concomitantly improving gut health. However, several inherent limitations should be acknowledged. First, because the IBS-SSS was administered to the entire study population, the findings should be interpreted as reflecting self-reported IBS-related symptom burden rather than clinically confirmed IBS severity. Second, given the cross-sectional design, the temporal relationships among RPA, depressive symptoms, and IBS-related symptom severity could not be established, and reverse causation cannot be excluded. These relationships warrant further investigation in longitudinal studies with temporally separated assessments. Third, the core study population consisted solely of university students from Shandong Province, China. This relatively homogeneous sample source resulted in limitations to the study’s scope, thereby affecting the generalizability of the findings to some extent. Finally, unmeasured confounding factors (e.g., genetic susceptibility, gut microbiota differences) present in the study may have influenced the experimental outcomes to some extent.

## 5 Conclusion

In summary, it was observed that RPA was significantly associated with lower IBS-related symptom burden in college students, and depressive symptoms statistically accounted for part of these associations. These findings emphasize the potential relevance of lifestyle factors to functional gastrointestinal symptoms. Novel insights were provided into the associations between physical activity, emotional well-being, and gastrointestinal function, which may offer new perspectives on non-pharmacological approaches for managing IBS-related symptoms.

## Data Availability

The raw data supporting the conclusions of this article will be made available by the authors, without undue reservation.
