# Supplementary material for: Depressive symptoms as a mediator in the association between regular physical activity and irritable bowel syndrome-related symptom severity: a cross-sectional study among college students
Source: Front Psychol. 2026 Sep 15;17:1856261. doi: 10.3389/fpsyg.2026.1856261 (PMC13619916; doi:10.3389/fpsyg.2026.1856261)
Supplement: Supplementary file 1 [file Supplementary_file_1.docx]

**Supplementary**

**1.** **Inclusion and exclusion Criteria**

The inclusion criteria were:

- Aged 18 - 30 years;
- Currently enrolled as a junior college, undergraduate, or graduate student;
- Voluntarily agreed to participate;
- Capable of independently completing the questionnaire, understanding the research content, and cooperating with the survey process.

The exclusion criteria were:

- Age outside the 18 - 30 range;
- Abnormal questionnaire completion time (less than 3 minutes or more than 20 minutes);
- Highly concentrated response patterns;
- Responses inconsistent with reality or common sense;
- Physical discomfort preventing participation or failure to meet other study criteria.

**Table S1** Distribution of IBS-related symptom severity across participant characteristics (n = 872).

| **Variables** |  | **IBS-related symptom severity** | | | |
| --- | --- | --- | --- | --- | --- |
|  | **n** | **No/minimal**  **(n%)** | **Mild**  **(n%)** | **Moderate (n%)** | **Severe**  **(n%)** |
| **Gender** |  |  |  |  |  |
| Male | 264 | 141 (53.4) | 92 (34.8) | 28 (10.6) | 3 (1.1) |
| Female | 608 | 248 (40.8) | 310 (51.0) | 49 (8.1) | 1 (0.2) |
| **Age (years)** |  |  |  |  |  |
| < 20 | 84 | 39 (46.4) | 38 (45.2) | 6 (7.1) | 1 (1.2) |
| 20 – 25 | 753 | 331 (44.0) | 351 (46.6) | 68 (9.0) | 3 (0.4) |
| ≥ 25 | 35 | 19 (54.3) | 13 (37.1) | 3 (8.6) | 0 (0.0) |
| **Educational level** |  |  |  |  |  |
| College student | 463 | 234 (50.5) | 189 (40.8) | 36 (7.8) | 4 (0.9) |
| University student | 360 | 132 (36.7) | 192 (53.3) | 36 (10.0) | 0 (0.0) |
| Postgraduate student | 49 | 23 (46.9) | 21 (42.9) | 5 (10.2) | 0 (0.0) |
| **BMI (kg/m^2^)** |  |  |  |  |  |
| < 18.5 | 116 | 60 (51.7) | 50 (43.1) | 6 (5.2) | 0 (0.0) |
| 18.5 – 24.0 | 547 | 228 (41.7) | 264 (48.3) | 51 (9.3) | 4 (0.7) |
| 24.0 – 28.0 | 137 | 59 (43.1) | 66 (48.2) | 12 (8.8) | 0 (0.0) |
| ≥ 28.0 | 72 | 42 (58.3) | 22 (30.6) | 8 (11.1) | 0 (0.0) |
| **Domicile** |  |  |  |  |  |
| Rural | 586 | 269 (45.9) | 259 (44.2) | 55 (9.4) | 3 (0.5) |
| Urban | 286 | 120 (42.0) | 143 (50.0) | 22 (7.7) | 1 (0.3) |
| **Amount of sleep (hours)** |  |  |  |  |  |
| < 4 | 5 | 2 (40.0) | 2 (40.0) | 1 (20.0) | 0 (0.0) |
| 4 – 6 | 76 | 25 (32.9) | 41 (53.9) | 8 (10.5) | 2 (2.6) |
| 6 – 8 | 673 | 305 (45.3) | 310 (46.1) | 56 (8.3) | 2 (0.3) |
| ≥ 8 | 118 | 57 (48.3) | 49 (41.5) | 12 (10.2) | 0 (0.0) |
| **Smoking habit** |  |  |  |  |  |
| No | 841 | 373 (44.4) | 390 (46.4) | 74 (8.8) | 4 (0.5) |
| Yes^a^ | 31 | 16 (51.6) | 12 (38.7) | 3 (9.7) | 0 (0.0) |
| **Passive smoking**^b^ |  |  |  |  |  |
| < 1 time a week | 474 | 239 (50.4) | 194 (40.9) | 38 (8.0) | 3 (0.6) |
| 1 – 3 times a week | 325 | 123 (37.8) | 172 (52.9) | 30 (9.2) | 0 (0.0) |
| 4 – 6 times a week | 50 | 15 (30.0) | 30 (60.0) | 5 (10.0) | 0 (0.0) |
| ≥ 7 times a week | 23 | 12 (52.2) | 6 (26.1) | 4 (17.4) | 1 (4.3) |
| **Alcohol consumption** |  |  |  |  |  |
| Never | 692 | 312 (45.1) | 324 (46.8) | 54 (7.8) | 2 (0.3) |
| < 2 times a week | 167 | 74 (44.3) | 74 (44.3) | 17 (10.2) | 2 (1.2) |
| ≥ 2 times a week | 13 | 3 (23.1) | 4 (30.8) | 6 (46.2) | 0 (0.0) |
| **Spicy food consumption** |  |  |  |  |  |
| Never | 98 | 50 (51.0) | 36 (36.7) | 10 (10.2) | 2 (2.0) |
| < 2 times a week | 358 | 165 (46.1) | 164 (45.8) | 28 (7.8) | 1 (0.3) |
| 3 – 5 times a week | 311 | 134 (43.1) | 149 (47.9) | 28 (9.0) | 0 (0.0) |
| ≥ 6 times a week | 105 | 40 (38.1) | 53 (50.5) | 11 (10.5) | 1 (1.0) |
| **Yoghurt consumption** |  |  |  |  |  |
| Never | 146 | 67 (45.9) | 64 (43.8) | 14 (9.6) | 1 (0.7) |
| < 2 times a week | 520 | 236 (45.4) | 239 (46.0) | 42 (8.1) | 3 (0.6) |
| 3 – 5 times a week | 181 | 80 (44.2) | 84 (46.4) | 17 (9.4) | 0 (0.0) |
| ≥ 6 times a week | 25 | 6 (24.0) | 15 (60.0) | 4 (16.0) | 0 (0.0) |
| **Takeaway consumption** |  |  |  |  |  |
| Never | 273 | 140 (51.3) | 114 (41.8) | 19 (7.0) | 0 (0.0) |
| < 2 times a week | 451 | 200 (44.3) | 215 (47.7) | 33 (7.3) | 3 (0.7) |
| 3 – 5 times a week | 102 | 40 (39.2) | 47 (46.1) | 15 (14.7) | 0 (0.0) |
| ≥ 6 times a week | 46 | 9 (19.6) | 26 (56.5) | 10 (21.7) | 1 (2.2) |
| **Sugar-sweetened beverage consumption** |  |  |  |  |  |
| Never | 131 | 67 (51.1) | 53 (40.5) | 10 (7.6) | 1 (0.8) |
| < 2 times a week | 506 | 224 (44.3) | 240 (47.4) | 40 (7.9) | 2 (0.4) |
| 3 – 5 times a week | 191 | 81 (42.4) | 92 (48.2) | 18 (9.4) | 0 (0.0) |
| ≥ 6 times a week | 44 | 17 (38.6) | 17 (38.6) | 9 (20.5) | 1 (2.3) |
| **Fruit consumption** |  |  |  |  |  |
| Never | 45 | 19 (42.2) | 18 (40.0) | 7 (15.6) | 1 (2.2) |
| < 2 times a week | 351 | 153 (43.6) | 173 (49.3) | 24 (6.8) | 1 (0.3) |
| 3 – 5 times a week | 361 | 155 (42.9) | 168 (46.5) | 36 (10.0) | 2 (0.6) |
| ≥ 6 times a week | 115 | 62 (53.9) | 43 (37.4) | 10 (8.7) | 0 (0.0) |
| **Frequency of physical activity** |  |  |  |  |  |
| < 1 time a week | 246 | 89 (36.2) | 126 (51.2) | 29 (11.8) | 2 (0.8) |
| 1 – 2 times a week | 246 | 107 (43.5) | 119 (48.4) | 18 (7.3) | 2 (0.8) |
| 3 – 4 times a week | 228 | 109 (47.8) | 98 (43.0) | 21 (9.2) | 0 (0.0) |
| 5 – 7 times a week | 128 | 68 (53.1) | 53 (41.4) | 7 (5.5) | 0 (0.0) |
| ≥ 8 times a week | 24 | 16 (66.7) | 6 (25.0) | 2 (8.3) | 0 (0.0) |
| **Duration of each physical activity (minutes)** |  |  |  |  |  |
| < 15 | 264 | 100 (37.9) | 133 (50.4) | 30 (11.4) | 1 (0.4) |
| 15 – 30 | 354 | 149 (42.1) | 175 (49.4) | 28 (7.9) | 2 (0.6) |
| 30 – 60 | 169 | 93 (55.0) | 65 (38.5) | 10 (5.9) | 1 (0.6) |
| 60 – 90 | 64 | 34 (53.1) | 23 (35.9) | 7 (10.9) | 0 (0.0) |
| ≥ 90 | 21 | 13 (61.9) | 6 (28.6) | 2 (9.5) | 0 (0.0) |
| **Symptoms after physical activity** |  |  |  |  |  |
| LCBH | 169 | 75 (44.4) | 71 (42.0) | 22 (13.0) | 1 (0.6) |
| BHSUS | 489 | 222 (45.4) | 230 (47.0) | 35 (7.2) | 2 (0.4) |
| FBH | 172 | 73 (42.4) | 84 (48.8) | 15 (8.7) | 0 (0.0) |
| RBH | 42 | 19 (45.2) | 17 (40.5) | 5 (11.9) | 1 (2.4) |

a: current smokers and past smokers were included; b: exposure to smoke caused by smokers for more than 15 minutes was counted as a single exposure; BMI: body mass index; LCBH: little change in breathing and heartbeat, usually no sweating; BHSUS: breathing and heartbeat sped up slightly, sweating slightly; FBH: fast breathing and heartbeat, sweating more; RBH: rapid breathing and heartbeat, sweating profusely.

**Table S2** Distribution of anxiety and depressive symptom severity among participants (n = 872).

| **Variables** | **Non (n%)** | **Mild (n%)** | **Moderate (n%)** | **Severe (n%)** |
| --- | --- | --- | --- | --- |
| Depression | 47.4 | 37.3 | 13.6 | 1.7 |
| Anxiety | 67.4 | 26.0 | 5.3 | 1.3 |

**Table S3** Associations of depressive and anxiety symptoms with gastrointestinal symptom severity (n = 872).

| **Exposure** | **Model 1** | | **Model 2** | |
| --- | --- | --- | --- | --- |
|  | ***β* (95% *CI*)** | ***P*** | ***β* (95% *CI*)** | ***P*** |
| **IBS-SSS score** |  |  |  |  |
| SAS score | 2.602 (2.176, 3.027) | < 0.001 | 2.458 (2.026, 2.890) | < 0.001 |
| SDS score | 1.927 (1.509, 2.343) | < 0.001 | 1.745 (1.318, 2.172) | < 0.001 |
| **GSRS score** |  |  |  |  |
| SAS score | 0.569 (0.489, 0.649) | < 0.001 | 0.549 (0.468, 0.629) | < 0.001 |
| SDS score | 0.415 (0.335, 0.494) | < 0.001 | 0.380 (0.299, 0.461) | < 0.001 |

Model 1 was unadjusted. Model 2 was adjusted for potential confounders. For IBS-SSS, adjusted models controlled for gender, educational level, sleep duration, and dietary habits (frequency of takeaway and sugar-sweetened beverage consumption). For GSRS, adjusted models controlled for gender, sleep duration, and dietary factors (spicy food, takeaway, and sugar-sweetened beverage consumption). *β*: regression coefficients; *CI*: confidence interval; SAS: self-rating anxiety scale; SDS: self-rating depression scale; GSRS: gastrointestinal symptom rating scale; IBS-SSS: irritable bowel syndrome symptom severity scale.

Figure S1 Diagnostic plots for the adjusted multiple linear regression model evaluating the association between RPA and IBS-SSS score.

The four panels show the residuals versus fitted values, normal Q-Q plot, scale-location plot, and residuals versus leverage plot, respectively. These diagnostic plots were used to assess linearity, approximate normality of residuals, homoscedasticity, and influential observations in the adjusted multiple linear regression model. The model was adjusted for sex, educational level, sleep duration, takeaway food consumption, and sugar-sweetened beverage consumption. IBS-SSS: irritable bowel syndrome symptom severity scale; RPA: regular physical activity.

Figure S2 Diagnostic plots for the adjusted multiple linear regression model evaluating the association between RPA and GSRS score.

The four panels show the residuals versus fitted values, normal Q-Q plot, scale-location plot, and residuals versus leverage plot, respectively. These diagnostic plots were used to assess linearity, approximate normality of residuals, homoscedasticity, and influential observations in the adjusted multiple linear regression model. The model was adjusted for sex, sleep duration, spicy food consumption, takeaway food consumption, and sugar-sweetened beverage consumption. GSRS: gastrointestinal symptom rating scale; RPA: regular physical activity.
